# The development of education for learners with diverse learning needs in the South African context: A bio-ecological systems analysis

**DOI:** 10.4102/ajod.v9i0.670

**Published:** 2020-02-10

**Authors:** Suegnet Smit, Lynn D. Preston, Johnnie Hay

**Affiliations:** 1 School of Psycho-Social Education, Faculty of Education Sciences, North-West University, Potchefstroom, South Africa; 2 School of Psycho-Social Education, Faculty of Education, North-West University, Vanderbijlpark, South Africa

**Keywords:** special education, challenges, diversity, inclusive education, bio-ecological systems analysis

## Abstract

**Background:**

Prior to 1994, special education in South Africa was marginalised and fragmented; therefore, the new democratic government promoted inclusive education as a means to transform education in general and diverse education in particular. However, transformation in diverse education is seemingly moving forward at a snail’s pace – too slow to benefit all learners experiencing barriers to learning and development.

**Objectives:**

This article serves a dual purpose: firstly, to apply a bio-ecological approach to highlight the historic development of diverse education and, secondly, to explore the interactive processes within the systemic levels in the South African education system, which affects the learner on the person dimension of the bio-ecological approach.

**Method:**

A document analysis approach was utilised to collect information by exploring a large body of research literature, which included academic articles, reports, policies and policy reviews. Data were categorised within the systems of the bio-ecological model to determine successes and challenges at each level.

**Results:**

Results from the bio-ecological systems analysis of related literature revealed not only many successes but also many challenges that inhibit change, growth and development in the South African education system, even more so for children experiencing barriers to learning.

**Conclusion:**

The transformation process of change from *what was* to *what should be*, regarding diverse education, seems to be stuck at *what is* and not moving forward to *what could be.* It has not transformed significantly enough to fill the gap between reality and the envisaged aim or dream of quality education for all.

## Introduction

Worldwide, the implementation of inclusive education (IE) has been problematic (Berlach & Chambers 2011) and South Africa (SA) is no exception. Education for learners with diverse educational needs, embedded within the inclusive model (Du Plessis 2013), is still not conforming to the expectations envisaged in the Education White Paper 6 (EWP6) (Department of Education [DOE] 2001) concerning equal education for all (Engelbrecht et al. 2016). By creating opportunities for effective learning, the constitutional right of every child of schoolgoing age in SA (Geldenhuys & Wevers 2013; Pillay & Di Terlizzi 2009), including learners with barriers to learning and development (DOE 2001), can be addressed.

The Department of Basic Education (DBE [formerly DOE]) states that its goal is to minimise, remove and prevent barriers to learning and development in the educational settings by attending to the unique needs of the individual learner (DOE 2001). This can be achieved by early identification and addressing the diverse needs of learners. However, the gap between reality and this ideal of IE cannot be bridged (Engelbrecht et al. 2016). Despite the commitment of the department to take responsibility to create equal opportunities for all learners (DOE 2001) and sustain effective learning in schools, general education remains poor (Donohue & Bornman 2014), with the process of change being slow (Reddy, Juan & Meyiwa 2013). In SA, as the world over, attempts to minimise exclusion are ineffective, resulting in exclusion being more evident than ever (Kaur & Arora 2014).

Twenty-four years into democracy, SA still cannot claim that all learners profit from quality education and service provision contrary to the vision of the government to correct inequalities (Dreyer 2017). The lack of appropriate service provision by the DBE impedes and obstructs its own set benchmarks for educational reform initiatives and generates even greater challenges (Du Toit & Forlin 2009). This situation evolves into what is described by Donohue and Bornman (2014:1) as a ‘crisis in education’, which influences the realisation of IE and jeopardises its success (Nel, Nel & Hugo 2012). This brings about the question that guided this research, namely, from a bio-ecological perspective: which successes and challenges contribute to the current state of education for learners experiencing barriers to learning?

## Methodology

The literature review included a search through academic articles, academic books, policies and reports on special education in SA, and IE in SA and the world over. Sources that did not address the history of special education, prior to and post-1994 diverse education, or the bio-ecological model, were eliminated.

Keywords used for the search included ‘special education in the South African context (and the world over)’, ‘special needs education’, ‘diversity in education’, ‘DBE policy documents’, ‘DBE reports’, ‘problems in special education’, ‘challenges in special education’, ‘IE’, and ‘implementation of IE’ – or a combination of the above-mentioned keywords.

### A bio-ecological systems perspective: A person system within a contextual system

The inclusive approach is ‘consistent with a systemic and developmental approach to understanding problems and planning action’ (DOE 2001:19). Bronfenbrenner’s bio-ecological process–person–context–time (PPCT) model (Bronfenbrenner & Morris 1998) provides a comprehensive framework reflecting both the systemic and developmental dimensions, making this model useful for the classification of phenomena related to the person–context interaction (Griffore & Phenice 2016). Thus, the PPCT model will serve as a theoretical framework based on which the various conceptions in the study can be explained and qualified. This framework facilitates the systemic explanation of the complex reciprocal interactions and proximal processes between the individual and the layers of systems involved in diverse education (Zimmerman & Kontosh 2007).

The *process* dimension in the PPCT model is at the core of the model and represents dual interactions between the *person* dimension (the individual) and the *context* dimension (the layers of environments) in a *time* dimension (a period of time). This core initiates and sustains human development. In the *process* dimension, the specific forms of interactions within the time period of the proximal processes have the capacity to directly or indirectly impact human development, resulting in the physical, biological, psychological, social and/or cultural development of the individual within systemic contexts (Bronfenbrenner & Morris 1998, 2006). Furthermore, internal and external reciprocal interacting factors between the processes of human development and the systems result in change, growth and development or stagnation, withering and underdevelopment for both the individual and the systems (Bronfenbrenner & Morris 1998). Proximal processes extend to multifaceted relationships and interactions between the individual’s personal and interpersonal processes, other individuals, objects or symbols. These proximal processes occur in the immediate environment and relate mostly to the *micro* environment (Swart & Pettipher 2016). For the processes to activate and sustain development (Bronfenbrenner & Ceci 1994), regular occurrence of specific events over a period of time is a prerequisite (Jackson et al. 2006).

**FIGURE 1:**
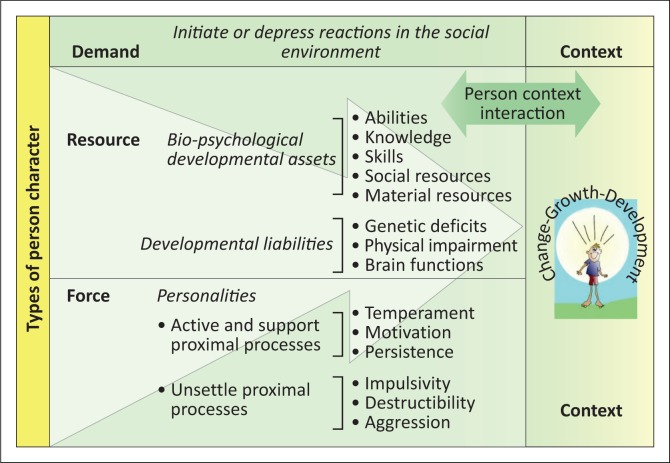
The application of the process–person–context–time in child development.

The capacities of the proximal processes are determined by personal inherent qualities or characteristics (see Figure 1), as well as both the direct and distant environments (Bronfenbrenner & Morris 1998, 2006). Personal inherent qualities, such as attributes of the individual, interact with each other. These qualities are determined by force, resource and demand characteristics that directly influence the proximal processes in order to either support or interrupt development. Force characteristics include aspects such as temperaments and personalities that activate and support proximal processes, for example, motivation and persistence, or characteristics that unsettle proximal processes, such as impulsivity, distractibility and aggression (Swart & Pettipher 2016; Zimmerman & Kontosh 2007).

In addition, force characteristics (locus of control and self-control) encompass the belief systems of the individual in relation to the larger systems (Jackson et al. 2006). Resource characteristics determine whether an individual is able to interact successfully during the proximal processes. Resources are defined as biopsychological developmental assets (abilities, knowledge, skills, experiences and social and material resources) or developmental liabilities (genetic deficits, physical impairments and damage to brain function) (Jackson et al. 2006). Demand characteristics are actions that initiate or depress reactions from the social environment (Swart & Pettipher 2016) to enable or interrupt proximal processes, for example, ‘age, gender, skin colour and physical appearance’ (Tudge et al. 2009:200).

The *context* dimension in the PPCT model refers to the direct and distant environmental levels that influence the learner’s life directly or indirectly and assist or constrain the proximal processes. Features such as political climate, policies and attitudes (Jackson et al. 2006) are good examples of influences on the proximal process regarding the context dimension. Levels in the direct and distant environments comprise the *micro, meso, exo* and *macro* systems that function within a *chrono* system.

The *micro* system includes the direct or immediate environment of the individual; the *meso* system embodies the linking interactions between one or more *micro* systems enclosing the developing individual. The *exo* system is the larger social system that involves the connections and processes between two or more settings where at least one does not encompass the developing individual (Bronfenbrenner & Ceci 1994). The *macro* system as the outermost layer comprises cultural values, customs and laws (Berk 2000). The environments function within the *time* dimension of the PPCT, capturing the changes and duration of the interactions between all the above-mentioned systems (Geldenhuys & Wevers 2013). This can be reflected in the changes in structure of education for learners experiencing barriers to learning.

Individuals and groups in different levels of environments link through changing, interdependent and interacting relationships (Onwuegbuzie, Collins & Frels 2013). The interdependence that exists between organisms and their physical environment should be considered holistically in order to understand how each system and sub-system contribute to the support and sustainment of the larger system (Donald, Lazarus & Moolla 2014). Events in one part of a system affect other systems, demonstrating the reciprocal nature of the relationships, often influencing the whole larger system (Bronfenbrenner & Ceci 1994).

### From special education (prior to 1994) to education for learners experiencing barriers to learning (post-1994)

History enables us to consider events from the past and to reveal their influence on the management of current capabilities or problems within the educational system. In addition, history also highlights the importance of the nature of social interactions between education and other systems, which creates a holistic approach to teaching and learning (Donald et al. 2014). Therefore, analysing the contextual factors of an education system assists in understanding the nature, structure and functioning of the education system in two historic timeframes (prior to and post-1994). Because a systems theory approach such as the PPCT model can accommodate changes in any dimension of the model (Zimmerman & Kontosh 2007), it has become the model of choice.

Contextual factors empower or prevent an educational system from ‘moving forward’. Contextual factors can, firstly, be historic in nature; secondly, relate to the communal relationships between layers in contexts; and, thirdly, concern the governance of an educational system (Steyn et al. 2017). Although the bio-ecological model was not a consideration in education practices before 1994, the social model of disabilities (Bronfenbrenner 1979) gained recognition; however, it lacked recognition of the *person* characteristics (Swart & Pettipher 2016). The discussion of both the pre- and post-apartheid era education for learners experiencing barriers will be based on the PPCT perspective to reveal the effect on the learner within the *person* dimension. The two eras in history represent different *time* dimensions in the history of the education system in SA, specifically education for learners experiencing barriers to learning. With the move towards the inclusive model, the applicability of the PPCT model (Bronfenbrenner & Morris 1998) is pertinent in explaining the successes and challenges that contributed to the current state of education for learners with diverse educational needs.

For the purpose of this article, the *person* dimension is reflected in the learner experiencing barriers, requiring diverse education. The *context* dimension represents the environmental levels in which the education system and sub-systems, directly or indirectly, influence the education of the learner, assisting or constraining proximal processes.

### Special education prior to 1994 viewed from a bio-ecological systems perspective

From a PPCT perspective, considering the reciprocal interactive proximal processes between the *perso*n dimension and the *context* dimension for the *macro* and *exo* levels (Bronfenbrenner & Morris 2006), there was no equality in the education system. Past laws and legislations were marginalising and discriminatory (Engelbrecht 2018), limiting or erasing any forms of equality.

Prior to 1994, the education system in SA was characterised by inequity, separate development, fragmentation, lack of transparency and lack of clarity in policy (DOE 1997). Furthermore, the ideology and belief system of that era influenced and mirrored inequality in the systemic layers (Rosa & Tudge 2013) of the South African education system.

In 1910, when the Union of SA was founded, no uniform national education system existed and each province (*exo* level) had its own education system. At this *exo* level, fragmented education departments, the lack of support provision and the lack of education in the mother tongue resulted in the exclusion of learners and for learners with barriers to learning to an even greater extent (DOE 1997). According to the constitution of that time, the medium of instruction was Dutch, which was later replaced with Afrikaans and English. Education reached only few learners of the black population (Fataar 1997) and ignored the African culture (Mphahlele & Mminele 1997 cited in Steyn et al. 2017). Moreover, the social context of separate development and the exclusion of learners led to limited access to support and resources for many (Pillay & Di Terlizzi 2009), and also isolation, bringing about contextual disadvantage and multiple social problems (Donald et al. 2014; Dreyer 2015). This influenced the interactions between *micro* systems in the *meso* system within the South African educational system.

At the *exo* level, special education (SE) was overseen by each provincial education department (Steyn et al. 2017). This resulted in a fragmented education system exacerbated by uneven access to SE and distribution of resources (DOE 1997).

In the *meso* system, both psychosocial features and physical context contribute to underlying proximal processes (Krishnan 2010) between the *micro* systems involved with the learner. The absence of the learner from school (*micro*), because of exclusion and/or placement policies (*macro* and *exo*), meant that the school experience, as an agent in proximal processes, was non-existent. This inhibited factors such as ability, experience, knowledge and skills development in a school environment (*micro*) from contributing to interactive proximal processes (Bronfenbrenner & Morris 1998, 2006). Engeström (2016) explains in the *activity-theoretical approach* to developmental research that the lack of mediation of mental processes in the teaching and learning environment restricts conveyance and increase of knowledge and skills, leading to undesired outcomes.

Furthermore, the ‘disability’ versus ‘normal’ classification according to the medical deficit model (Swart & Pettipher 2016) labelled learners. The term ‘disability’ (Swart & Pettipher 2016) had a narrow scope of SE needs because the environmental influences as contributing factors to barriers to learning were not considered. White learners with special needs had access to more specialised interventions and better resources, resulting in the neglect of providing support services to the majority of the black population (DOE 1997). This situation of separate development and support in SE continued until 1994 (Steyn et al. 2017).

Accepting that the medical model matched the era, the move towards the social model highlighted the fact that the first mentioned model did not adequately consider the cultural, social, economic, political and psychological systems’ influences on the individual (Nel 2013). When viewed from a PPCT perspective, the medical model falls short of demonstrating the complex reciprocal interactions and interrelationships (Swart & Pettipher 2016) that take place in the contexts (extrinsic barriers) of the education system by focussing only on the intrinsic barriers of the learner. In a ‘one fits all’ approach to remedial intervention (Du Plessis 2013), personal and interpersonal processes (Jackson et al. 2006) (see Figure 1) were not fully explored; therefore, some learners did not develop to their full potential. This past approach contradicts the current holistic view to intervention (Department of Basic Education [DBE] 2014), where support is individualistic (the individual support programme [ISP]) and uniquely tailored.

Considering the school as the *micro* level, the medical model of disability based evaluations on medical testing (Ferguson 2008). Medical professionals, therapists, specialists and remedial teachers provided remedial intervention (Pillay & Di Terlizzi 2009).

Furthermore, teaching and learning were teacher-centric, using direct teaching methods (Schunk 2012). Interventions highlighted what the learner lacked and did not concentrate on the *person* or *context* characteristics and/or assets (King & Madsen 2007), or learner strengths, advocated by EWP6 (DOE 2001).

Bronfenbrenner and Morris (2006) state that human development should not only be perceived objectively, but also include an experiential or activity element (Engeström 2016), whereby the learner perceives the environment through personal feelings or opinions, resulting in the learner becoming an active participant in his or her own development. Thus, the learner should have been participating in constructing the *micro* environment through *person–context* interactions (Bronfenbrenner & Morris 2006). Yet, in past cases, where the personal attributes of the learner were ignored, retaining the learner and keeping the learner dependent (Maguvhe 2015) on other individuals and systems resulted in slowing down or the stagnation of ‘*change*’, ‘*growth*’ and ‘*development*’. In turn, the development of *person* characteristics was constrained and *person–context* interactions gradually became dysfunctional (Bronfenbrenner & Evans 2000). This implies that the past education system did not consider a holistic approach in addressing learners’ needs and although there was development of the *person*, the *person–context* interaction was overlooked, which led to the deprivation of human and social development of the learner (Bronfenbrenner & Evans 2000).

### Post-1994 education for learners experiencing barriers to learning

Considering the historical influences up to 1994, education for learners experiencing barriers is portrayed as being of poor quality, specifically for the disadvantaged population (Daniels 2010). The outcomes of these historic events led to transformation, changing laws, policies and structures (Steyn et al. 2017). The expectation that the democratic government would provide better living conditions for a larger part of the population, in line with the basic human rights principle (Fataar 1997), endorsed an anticipation that with the move away from apartheid education, education for learners experiencing barriers would flourish within the inclusive paradigm.

Based on the findings of the National Education Policy Investigation (NEPI) (National Education Coordinating Committee [NECC] 1993) and the value framework of democracy, suggestions were made on policy issues concerning areas of education related to support services at the *macro* level (NECC 1993).

The transition to IE unlocked a new value system of inclusion, opening new opportunities (Dalton, McKenzie & Kahonde 2012) for the education of learners experiencing barriers, yet the implementation thereof remains problematic (Daniels 2010; Engelbrecht 2018). A notable mind-shift implied that the individual no longer has to ‘fit into’ the educational system, but that the educational system needs to adapt to meet the needs of the individual child (Kaur & Arora 2014). Intervention now focussed on learner strengths and capabilities, considering the contextual influences (King & Madsen 2007). The NEPI recommendations had an extensive influence on adaptation of the education system at *macro* and *exo* levels, resulting in the drafting of policies and a commendable number of guideline documents (DOE 1997), such as the EWP6 (DOE 2001); the conceptual and operational guidelines for the implementation of IE: full-service or inclusive schools (DOE 2005a) and guidelines for support schools as resource centres (DOE 2005b); and the national strategy on screening, identification, assessment and support (SIAS) (DBE 2014) which outlines the implementation of EWP6.

The target actions of EWP6 (DOE 2001) outline activities for education transformation to improve the quality of education. The government’s intentions to place high priority on the minimisation, removal and prevention of barriers to learning and development in the educational context by means of prioritising the restructuring and improvement of education support services (DOE 1997; Du Plessis 2013) were highly supported. The SIAS policy (DBE 2014) serves as a framework for procedural standardisation to screen, identify, assess and provide ISPs for all learners, specifically vulnerable learners who require additional support to increase their participation and inclusion in the school. Over and above a strategic plan to implement IE (DOE 2001), adapting the curriculum to provide for the specific needs of the learners through the availability of a safe and supportive learning environment was widely supported (DOE 1997). To support the move away from the segregation and marginalisation of learners with diverse educational needs and the strive towards inclusion for all, EWP6 (DOE 2001) and SIAS (DBE 2014) stipulate that learners must be assessed and placed in accordance with the level and nature of support needed and not placed in schools according to categorisation of the type of disability, as was previously the norm.

### The reality of the current education system for learners experiencing barriers to learning from a process–person–context–time perspective

**FIGURE 2:**
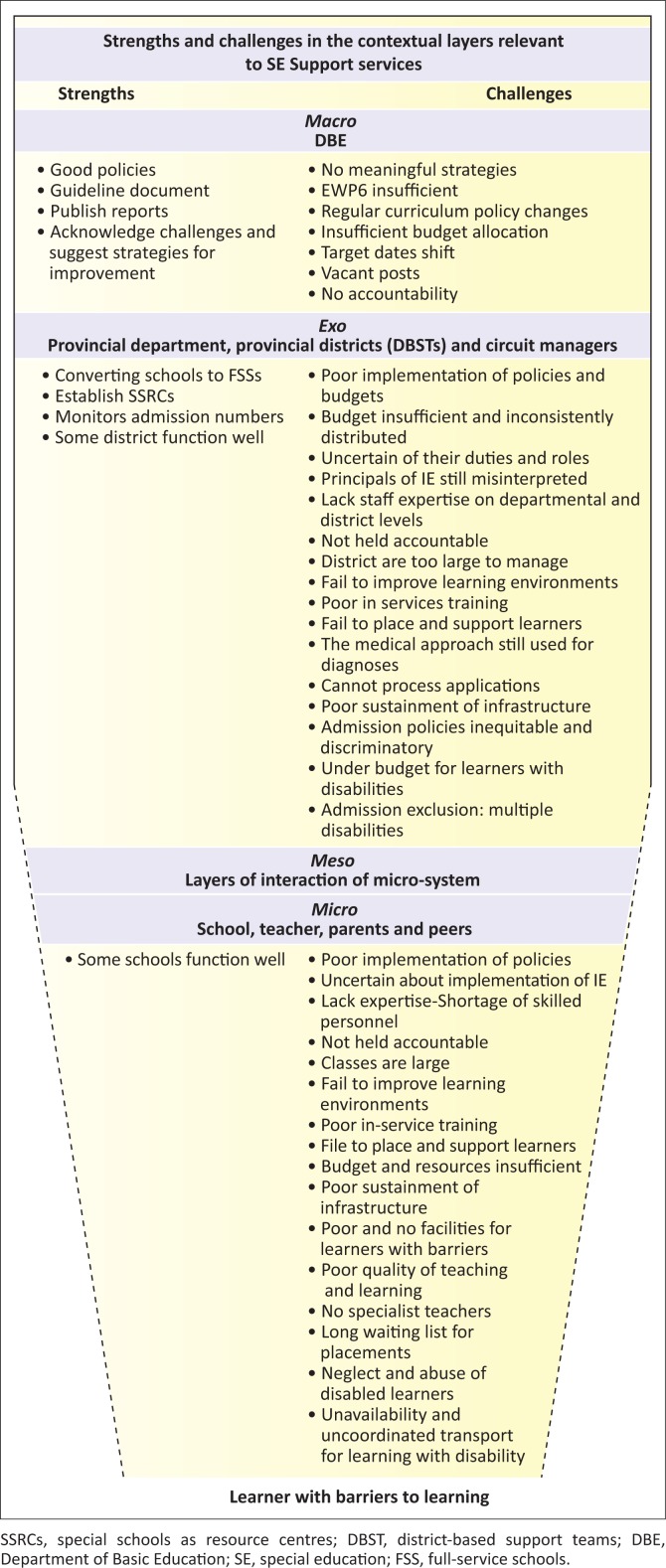
Learner with barriers to learning.

The layers of systems and parts within each layer – according to the PPCT perspective – provide a holistic picture of the functioning of the education system, as illustrated in Figure 2. A closer look at the interrelationships between systemic layers and governance reveals the strengths and challenges contributing to the problematic implementation of IE and education for learners with barriers in all the system layers (King & Madsen 2007).

Challenges are experienced in all the system layers, but then even more alarming are the consequences of the interplay between *person–context* and the resulting outcomes that seep down between the layers of systems to the learner in the classroom, affecting child development negatively, exactly what Chapter 1, number 1.5.3 in EWP6 (DOE 2001) does not advocate. Acknowledging the influence of legacies of the previous dispensation, the factors that empower or constrain communal relationships in the current educational context and governance of the educational system are highlighted from the PPCT perspective to make suggestions as to why education for learners experiencing barriers to learning has not moved forward more rapidly.

The DBE as a *macro* system portrays the ideologies and value system of IE (Nel 2013), representing the larger governing system of education responsible for laws, legislation, policy drafting, strategic planning, coordination of planning and funding for education for learners experiencing barriers to learning (Daniels 2010; DBE 2012a).

Top-down governance, starting at the distal *macro* system, subsequently determines the outcomes of interactions in the system layers to portray the successes and challenges of the governing system (Steyn et al. 2017). Furthermore, embedded in IE, each system and sub-system network has to initiate interactive processes with the *person* (learner experiencing barriers to learning) who is functioning as a system within the education system (Swart & Pettipher 2011).

An encouraging achievement is that the DBE is reaching the target for converting ordinary schools to full-service schools (FSSs) and establishing special schools as resource centres (SSRCs), which resulted in the removal of segregation, enabling more children to attend school than pre-1994. Although the department regularly publishes progress reports in which they acknowledge weaknesses and, in many cases, suggest strategies to minimise the challenges (DBE 2015a, b) there are still too many eminent challenges (see Figure 2). There are still too many children with barriers or disabilities who are not yet accommodated in any school because admission policies for ordinary and SSs remain inequitable and, therefore, discriminatory. Infrastructure is also poor and the lack of knowledgeable personnel contributes to the problem of implementation (Department of Women Children & People with Disabilities [DWCPD] 2013).

The DBE is under serious criticism for the poor implementation of IE and it appears that education for learners experiencing barriers starts to disintegrate in the *macro* system, where the DBE controls the factors influencing and contributing to the current status. Therefore, I take the stance that the top-down governance causes the strengths and challenges to accumulate in each layer of the system (Figure 2), causing a ‘funnel’ effect downwards through the other systems to the learner-person system (Figure 2). This is based on the assumption that processes in one particular system do not necessarily cause challenges in that system alone (Swart & Pettipher 2011), but because of constant interactions between many systems, a circular feedback loop arises, creating the situation that currently exists.

The *exo* is an influential system in education, as it provides a bridging area between the nine provincial departments (the second layer of government) (Steyn et al. 2017) and the meso system. The departments liaise with district-based support teams (DBSTs) to function in accordance with national and provincial legislation (DBE 2010). The DBSTs include staff from provincial, regional districts and SSRCs (DOE 2001), directed by the decisions made in the *macro* system. The provincial departments and the DBSTs oversee and implement the strategic plans for support service provision to schools, for example, investment in whole school development by assisting and supporting principals (Mafuwane & Pitsoe 2014), teachers and learners (DOE 2001).

To further illustrate this systemic perspective, one must bear in mind that, understandably, the challenges already identified in the *macro* system create difficulties when the implementation of policies and budgets is executed at the *exo* level. For example, the policy on the organisation roles and responsibilities of education districts (DBE 2012b) contains a distressing remark: ‘there has been no common formulation of what a district education office should be or do’. The report confirms that only some district offices understand their roles, leading to a low level of efficiency of DBSTs. If this is the case, how can one expect the school sub-system to function effectively? Furthermore, some districts are too large to accommodate visitation and support to all schools (DBE 2012b). Considering this, there is no certainty that the district teams adequately contribute to improve learning environments for learners by refining the abilities of principals and teachers.

Collaboration (*process*) between systems is fundamental to the success of IE (Nel, Nel & Lebeloane 2016). Therefore, if there is insufficient collaboration between the *exo* and the *meso* systems, the *process* will be interrupted in the *meso* system, resulting in the education to learners with barriers being further fragmented because of lack of service provision and communication between the DBE, DBSTs and schools. Even though the DBE promised that ordinary schools, FSSs and SSRCs, in collaboration with DBSTs, would become solid support structures to learners with learning breakdown and disabilities (DOE 2005b), this is not evident in reality. It appears that not enough has been put in place concerning schooling in general, and education for learners with diverse learning needs in particular, to counteract the high proportion of repetition figures, school dropout percentage and quality of teaching and learning (Smit, Wood & Neethling 2015). There are still about 70% of children with disabilities not accommodated in schools (DOE 2005b), whether in ordinary schools, FSSs or SSRCs.

In the *meso* system, *micro* systems interact, acknowledging that the learner-person, as a developing individual (Bronfenbrenner & Ceci 1994), is concurrently involved with different *micro* systems (e.g. school, family, peers and community). *Meso* systems support developmental characteristics in the form of *processes* (teaching, interventions, learning and counselling) (Engeström 2016). Activities, roles and relationships taking place across settings (Rosa & Tudge 2013) emphasise exposure and active participation of the learners in these settings, resulting in learning experiences (experiential learning) (Rosa & Tudge 2013). When the learner is denied these opportunities to partake in activities, outcomes (the result of activities) are not reached. The effects of proximal *processes* (outcomes) can be more influential on the developing person than the interactions themselves (Bronfenbrenner & Ceci 1994), especially when we consider, for example, resilience (Pearson, Pearce & Kingham 2013) and coping skills (King & Madsen 2007). It should be noted that the dynamism of the proximal *processes* varies according to the *person* characteristics and the environmental *context* (Bronfenbrenner & Morris 2006).

The school is a *micro* setting where activities, interpersonal roles and relations should be supportive of developmental characteristics (Rosa & Tudge 2013). The learner is part of strengths and challenges in this setting, which will either protect or place the learner at risk (King & Madsen 2007). Teachers have an important role in reinforcing the development of *person* characteristics in the teaching and learning environment to influence *processes* for determining positive developmental outcomes. *Person* characteristics (e.g. a teacher or objects and/or symbols) direct the course and influence the *processes* (e.g. computers and textbooks) (Bronfenbrenner & Morris 2006:823). Force characteristics lead to ‘exploration, manipulation, elaboration and imagination’ (Bronfenbrenner & Morris 2006), which in turn all lead to progression in interactions underpinning learning (Evans 2003). The latter is hampered by aspects such as the lack of teacher expertise in education for learners requiring additional education or support (*person* factor); this may be because of poor qualifications and insufficient in-service training (Dreyer 2017) or the unavailability of resources and sustenance of infrastructure (DWCPD 2013).

Participation in *processes* develops the learner’s biological resources of ‘ability, motivation, knowledge and skills’ (Bronfenbrenner & Morris 2006) to participate in interactions with other persons in the school *context.* Within these supportive interactions, the learner builds independence (O’Toole, Hayes & Mhathúna 2014) and becomes a mediator and creator of his or her own development. However, in the absence of these resource characteristics, mediation does not take place, slowing down developmental outcomes (Bronfenbrenner & Morris 2006).

Considering this, I reason that the teacher, SBST and DBST play an important supportive role (Nel et al. 2016) in assisting the learner in developing resources. If this support is lacking, it will inhibit the onset and sustenance of force characteristics because demand characteristics attract or dampen interactions with the environment, which in turn nurture or interrupt the *processes* (Bronfenbrenner & Morris 2006). In the absence of force and biological resources, there is ineffective interaction from *person* to *context*. However, ineffective interaction from *context* to *person* contributes to the absence of force and biological resources (Tudge et al. 2009). In other words, the learner with barriers does not benefit from teaching and learning because the teacher has no experience in teaching or supporting the learner with barriers, resulting in the learner not being supported in strengthening of *person* characteristics. The opposite is also true – in case of stability of regular service provision, functional education structures and good physical environment, positive developmental outcomes as a dual *person*–*context* interactive relationship will occur. Even in disadvantaged schools, competent outcomes can outweigh dysfunction if stable environments are in place (Bronfenbrenner & Morris 2006) to support proximal *processes*.

I am of the opinion that because of the lack of accountability and optimal interaction between many of the DBE systems, a large gap is evident between the governing systems directly affecting the *person* system. As interactions between systems are of a reciprocal nature, many of the problems experienced at the *micro* level originate in the *macro* system, with other influences such as poor teaching and poor family circumstances, placing additional pressure on the support systems in the *micro* and *exo* systems. Although there are many other reasons why *micro* systems become dysfunctional, poverty and unemployment (Donald et al. 2014) to name a few, insufficient support systems (Engelbrecht, Oswald & Forlin 2006) are considered a major contributing factor in the school environment, in hampering effective teaching and learning. To add to this, insufficient school governance and poor teaching, especially in the rural areas (DWCPD 2013), are noted.

Bronfenbrenner and Morris (2006) emphasise in the PPCT model that the *process* of interaction between *person* and *context* for learning outcomes is more important than the *person* characteristics or the *context* factors viewed separately. Systemic assets or weaknesses from the *macro* and *exo* environments definitely create positive or negative interactive processes within the school environment, resulting in a ‘funnel effect’ regarding the problems directly channelled down to the learner. Acknowledging that the education system is not the only causal factor, I reason that instability and changes in the education system are influencing factors, directly or indirectly, in the *person–context process*, influencing developmental outcomes (Bronfenbrenner & Morris 2006).

## Conclusion

There is much blame, not without reason, attributed to *what was* in the South African educational system but it seems that too few lessons were learnt from the past mistakes, and *what should be* has not been realised. This article ventured into the terrain of many challenges with fewer achievements. Attempts to implement IE successfully in developed and developing countries are being noticed (Ahsan, Deppeler & Sharma 2013). The situation in SA (a developing country) clearly reflects the fact that the transformation process in education for learners experiencing barriers is progressing too slowly (Right to Education of Children with Disabilities Campaign [R2ECWD] 2016), even though the necessity of advancement to equalling international targets is the ideal (the Millennium Development Goal of universal primary education by 2015 and Education for All by 2015) (Kaur & Arora 2014). Nonetheless, the reality in SA suggests that the road to educational transformation has been a problematic and bumpy one, which by now should have had a greater and wider impact on learners experiencing barriers to learning and development (DBE 2011; SAHRC 2012). Furthermore, if the implementation strategy of IE (DOE 2001) had played a prominent role in the inclusion of all learners, a definite movement away from the conditions of SE before 1994 would have been more prominent. As IE has not optimally been realised in SA, there is evidence of many factors that still negatively affect the development of the *person* in the PPTC model. The lack of inclusive transformation ranges from scattered incidents of exclusion to situations of gross neglect regarding learners within the educational system.

## Recommendations

It would be unfair to compare development of education for learners experiencing barriers to learning in SA to other developed and leading countries (e.g. the United States of America). However, there is no harm in following these examples (Nel et al. 2012). Being a democratic country, the main objective of the education system should not be to merely provide education, but to provide quality education for all learners (DOE 2001; Du Plessis 2013). Simply providing a service does not make it accessible or worthwhile for at-risk children (DBE 2012a); thus, the focus must be on the teacher to bring changes to SE teaching and learning, and perhaps have more vigorous campaigning to make changes in the *macro* and *exo* education systems, setting the stage for quicker change and encouraging people to take individual responsibility.

In reflecting on education for learners experiencing barriers to learning in SA, I firstly suggest that better and closer collaboration between the DBE and higher educational institutions could benefit SE, in the sense that more focussed specialised teacher training could be provided by these higher education institutions, resulting in enhanced quality of teaching and learning. Secondly, inclusion of learners and the success of SE services are dependent on the functionality and effectiveness of the support systems and, therefore, transformation and change in education can only be achieved if the full range of education and training support services are provided and aligned (DOE 2001) and work together to pool their resources. This must be initiated from the bottom-up and the top-down all the way through to all the bio-ecological system layers within the PPCT model, ensuring that diverse education speedily moves from *what was* to *what should be*, and is freed from what is and moves to *what could be.*
